# The ZjMYB44-ZjPOD51 module enhances jujube defense response against phytoplasma by upregulating lignin biosynthesis

**DOI:** 10.1093/hr/uhaf083

**Published:** 2025-03-19

**Authors:** Liman Zhang, Hongtai Li, Ximeng Wei, Yuanyuan Li, Zhiguo Liu, Mengjun Liu, Weijie Huang, Huibin Wang, Jin Zhao

**Affiliations:** College of Life Science, Hebei Agricultural University, Baoding 071000, China; Hebei Key Laboratory of Plant Physiology and Molecular Pathology, Hebei Agricultural University, Baoding 071000, China; College of Life Science, Hebei Agricultural University, Baoding 071000, China; Hebei Key Laboratory of Plant Physiology and Molecular Pathology, Hebei Agricultural University, Baoding 071000, China; College of Life Science, Hebei Agricultural University, Baoding 071000, China; Hebei Key Laboratory of Plant Physiology and Molecular Pathology, Hebei Agricultural University, Baoding 071000, China; Key Laboratory of Insect Developmental and Evolutionary Biology, CAS Center for Excellence in Molecular Plant Sciences, Shanghai Institute of Plant Physiology and Ecology, Chinese Academy of Sciences, Shanghai 200032, China; Research Center of Chinese Jujube, Hebei Agricultural University, Baoding 071000, China; Research Center of Chinese Jujube, Hebei Agricultural University, Baoding 071000, China; Key Laboratory of Insect Developmental and Evolutionary Biology, CAS Center for Excellence in Molecular Plant Sciences, Shanghai Institute of Plant Physiology and Ecology, Chinese Academy of Sciences, Shanghai 200032, China; College of Life Science, Hebei Agricultural University, Baoding 071000, China; Hebei Key Laboratory of Plant Physiology and Molecular Pathology, Hebei Agricultural University, Baoding 071000, China; College of Life Science, Hebei Agricultural University, Baoding 071000, China; Hebei Key Laboratory of Plant Physiology and Molecular Pathology, Hebei Agricultural University, Baoding 071000, China

## Abstract

Lignin is a major component of the plant cell wall and has a conserved basic defense function in higher plants, helping the plants cope with pathogen infection. However, the regulatory mechanism of lignin biosynthesis in plants under phytoplasma stress remains unclear. In this study, we reported that *peroxidase 51* (*ZjPOD51*), which is involved in lignin monomer polymerization, was induced by phytoplasma infection and that overexpression of *ZjPOD51* in phytoplasma-infected jujube seedlings and *Arabidopsis* plants significantly increased their defense response against phytoplasma. Yeast one-hybrid (Y1H) and luciferase (LUC) assays showed that *ZjPOD51* transcription was directly upregulated by *ZjMYB44*. Genetic validation demonstrated that *ZjMYB44* expression was also induced by phytoplasma infection and contributed to lignin accumulation, which consequently enhanced phytoplasma defense in a *ZjPOD51*-dependent manner. These results demonstrated that the ZjMYB44-*ZjPOD51* module enhanced the jujube defense response against phytoplasma by upregulating lignin biosynthesis. Overall, our study first elucidates how plants regulate lignin to enhance their defense response against phytoplasma and provides clues for jujube resistance breeding.

## Introduction

Phenylpropanoid metabolism is a significant secondary pathway in plants, crucial for producing defense precursors against biological stress [1]. Lignin produced by this metabolism is vital for providing mechanical strength to cell walls and acting as a defensive barrier against pathogens [2, 3]. By depositing lignin in infected vessels, the spread of pathogens to surrounding areas is restricted, aiding in their containment and elimination at the infection site [4]. In *Arabidopsis*, lignin monomer polymerization occurs in highly restricted areas around pathogen cells, where local lignification around the pathogen reduces *Pseudomonas syringae* movement [5]. In grape, lignin deposition has been shown to be a limiting factor for the spread of pathogens between xylem vessels [6]. The resistance of tomato to *Ralstonia solanacearum* requires the deposition of a lignin cork coating [7]. These findings indicate that lignin is a very important and effective substance in plant defense against pathogens.

In dicotyledonous plants, the lignin monomers mainly include coniferyl and sinapyl alcohol [8], and the polymerization of lignin monomers occurs in the cell wall under the action of peroxidase (POD) or laccase [9, 10]. In *Arabidopsis*, loss of *AtPOD2* or *AtPOD25* function results in a decrease in lignin content [11]. Class III PODs serve an important role in the innate defense of plants against bacterial and fungal pathogens by mediating both passive and active defensive responses [12, 13]. Among plant defense mechanisms, rapid reactive oxygen species (ROS) generation is one such exemplar defense tactic that causes O_2_^.-^ generation and H_2_O_2_ production in apoplasts, and H_2_O_2_ is strictly regulated by Class III PODs, which serve as both producers and scavengers, depending on whether the enzyme is involved in peroxidative or hydroxylic cycles [14, 15]. Enhanced Class III POD activity was detected in leaves infected with yellow mosaic virus, and enzymatic antioxidants modulated the production of ROS to respond to pathogen infection [16]. In sweet orange *CsPOD25-OE* lines, tolerance to citrus bacterial canker was improved due to ROS homeostasis accompanied by elevated levels of H_2_O_2_ and increased lignification of the apoplastic barrier [17].

Lignin biosynthesis is regulated by a complicated transcriptional regulatory network [18]. POD is also regulated by multiple transcription factors (TFs), including MYB TFs. *OsMYB55/61* positively confers jasmonic acid (JA)-mediated *Xoo* resistance by upregulating the expression of *OsPOD26* to produce lignin [19]. *AtMYB44* contributes to a range of biotic stress responses, including resistance to *Myzus persicae* and *Plutella xylostella*, through the activation of *EIN2* expression [20]. And, *AtMYB44* acts as an integrator of crosstalk between JA and salicylic acid (SA) in plant defense responses [21]. However, little information is available on whether *MYB44* transcriptionally regulates lignin biosynthesis.

Jujube witches’ broom (JWB) is a typical phytoplasma disease characterized by symptoms such as yellowing, small dense leaves on clustered branches, phyllody, and no defoliation in winter [22]. Phytoplasma is a kind of prokaryote without cell wall that parasitizes plant phloem tissue [23, 24] and can widely spread by insect carriers such as leafhoppers [25, 26]. The technical limitation that the phytoplasmas cannot be cultured *in vitro* has limited the study of the jujube defense response against phytoplasma. Our previous study showed that POD activity in diseased trees in the early stage of phytoplasma infection was significantly greater than that in healthy ones and was also activated earlier in resistant trees than in susceptible ones [27]. However, the molecular regulatory network of lignin biosynthesis in jujube plants under phytoplasma stress remains unclear. In this study, *ZjPOD51* was first identified and verified to be involved in the defense response against phytoplasma. Furthermore, ZjMYB44 was screened as an upstream regulator. Thus, the molecular mechanism by which ZjMYB44-*ZjPOD51* mediates lignin synthesis to enhance the jujube defense response against phytoplasma was revealed. These results also provide novel insights into the prevention and control technology development to phytoplasma diseases.

## Results

### Phenylpropanoid biosynthesis was upregulated in jujube after phytoplasma invasion

**Figure 1:**
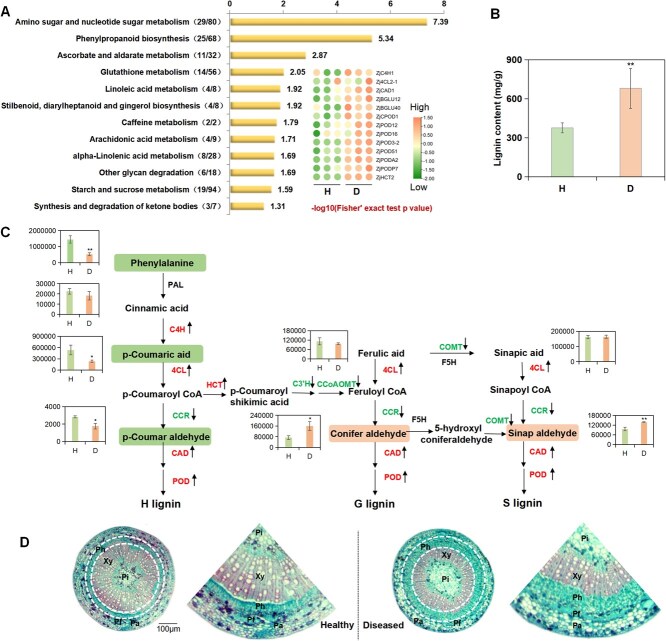
The lignin biosynthesis in jujube phloem was enhanced after phytoplasma infection. (**A**) KEGG pathway of DEPs in the phloem of healthy (H) and diseased (D) jujube trees. The heatmap represents the DEPs involved in lignin biosynthesis. The numbers in parentheses represent the number of mapping DEPs/the number of background proteins in this pathway. (**B**) The lignin contents in healthy (H) and diseased (D) jujube. (**C**) The changes of metabolites involved in lignin biosynthesis in healthy and diseased jujube (The bar chart shows the relative content of small-molecule metabolites). Bars represent the mean values ± SEs (*n* = 3). ^**^ represents *P* < 0.01, ^*^ represents *P* < 0.05. (**D**) Transverse structure of healthy and diseased jujube stems observed by paraffin section test. The arrow after the gene represents the change in gene expression level in the proteome (upward represents the increase, downward is decrease). Pa: parenchyma, Pf: phloem fiber, Ph: phloem, Xy: xylem, Pi: pith.

The proteomic data of the phloem of healthy and JWB-infected jujube trees were first analyzed. Kyoto Encyclopedia of Genes and Genomes (KEGG) pathway enrichment results revealed that 207 differentially expressed proteins (DEPs) were involved in 29 pathways. Among them, 12 pathways, including phenylpropanoid biosynthesis and amino sugar and nucleotide sugar metabolism, were significantly enriched (*P* < 0.05) (Fig. 1A). The phenylpropanoid metabolic pathway is the main pathway for plant secondary metabolites and plays a vital role in plant disease resistance and defense responses. Moreover, 25 DEPs were involved in the phenylpropanoid pathway (Table S1). Thus, phenylpropanoid metabolic pathway was considered as a potential regulator against phytoplasma invasion in jujube.

Lignin, as one of the important branch products of phenylpropanoid metabolism, plays a crucial role in plant defense against pathogens by increasing the thickness of the cell wall and regulating its plasticity, thereby limiting the invasion and movement of pathogens [17, 28, 29]. After phytoplasma infection, the lignin content in jujube phloem was significantly increased compared to the healthy one (Fig. 1B, Fig. S1A), especially the coniferyl and sinapyl aldehyde levels (Fig. 1C). Moreover, the stem microstructure showed that the proportion of phloem increased after phytoplasma infection (Fig. 1D), indicating that phloem development was stimulated and its lignin synthesis was also enhanced in diseased jujube.

### *ZjPOD51* might play important roles during phytoplasma invasion

**Figure 2:**
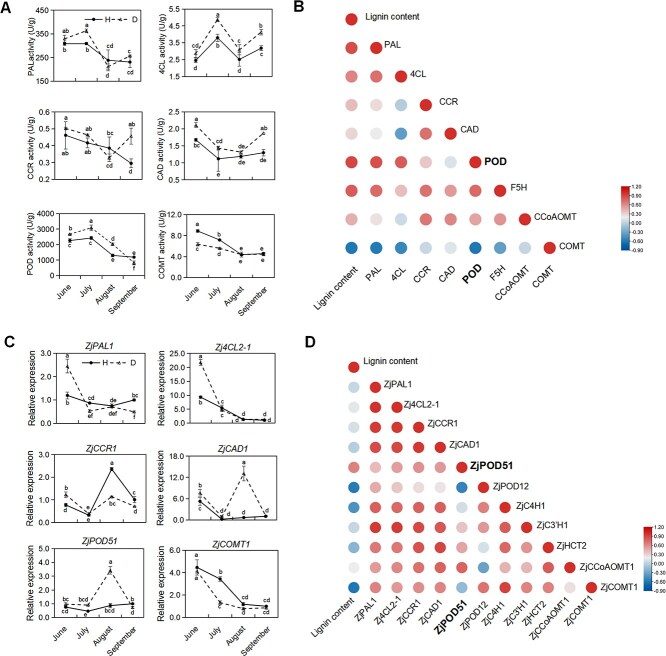
The changes of enzyme activities and gene expressions related to lignin biosynthesis in jujube during phytoplasma infection. (**A**) The activities of enzymes related to lignin biosynthesis in healthy and diseased jujube phloem. Duncan’s multiple range tests were used for statistical analysis, different letters (a, b, c, d...) indicate significant differences, while the same letters (such as a and a, ac and ab, ab and bc...) indicate no significant differences. (**B**) Correlation analysis between enzyme activity and lignin content. (**C**) The expressions of genes related to lignin biosynthesis in healthy and diseased jujube phloem. (**D**) Correlation analysis between gene expression and lignin content.

As shown in Fig. 2A and Fig. S2, the activities of most lignin synthesis-associated enzymes increased in the early stage of phytoplasma infection, and the activities of POD, 4-coumarate-CoA ligase (4CL), and cinnamyl alcohol dehydrogenase (CAD) increased during the whole period. POD activity was the highest, and the correlation between POD activities and lignin content was 0.89 (Fig. 2B). Moreover, *ZjPOD51* expression was greatly enhanced after phytoplasma infection and was strongly correlated with the lignin content (Fig. 2C and D). Therefore, *ZjPOD51* was selected as the candidate gene for further functional assays.

### ZjPOD51 is a Class III peroxidase

**Figure 3:**
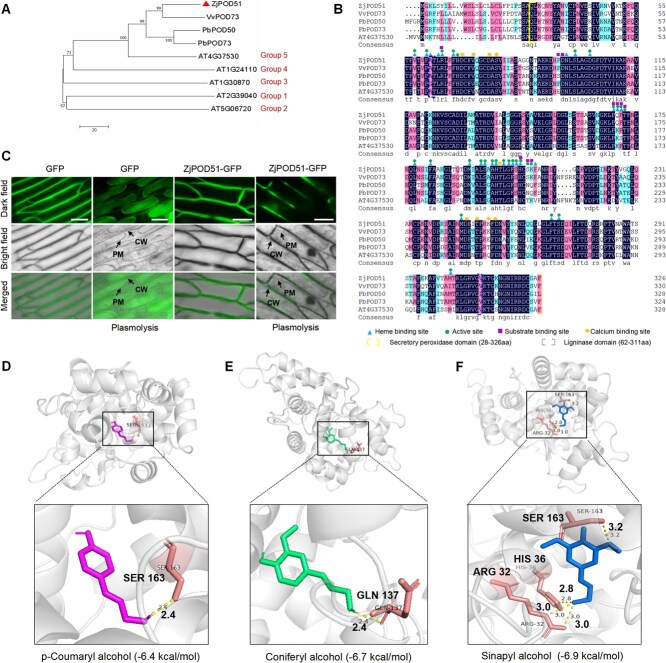
The phylogenetic analysis, subcellular localization, and molecular docking of ZjPOD51. (**A**) Phylogenetic analysis of ZjPOD51 and its orthologs of several other plants using the neighbor-joining method in MEGA7.0 with 1000 bootstrap iterations. The numbers at the nodes of the tree indicate the percentages of bootstrap values from 1000 replicates. Note: The triangle indicates ZjPOD51. VvPOD73 (*Vitis vinifera*), XP_002284278.1; PbPOD50 (*Pyrus bretschneideri*), XP_018500287.2; PbPOD73 (*P. bretschneideri*), XP_009342775.2; AT4G37530, AT1G24110, AT1G30870, AT2G39040, AT5G06720, five POD proteins of *A. thaliana*. (**B**) Multiple sequence alignment of ZjPOD51 and other homologous proteins. (**C**) Subcellular localization of ZjPOD51 in onion epidermis. Scale bar = 50 μm. The 0.3 g/ml sucrose solution was used for the plasmolysis experiment of onion epidermal cells. (**D**) Molecular docking between ZjPOD51 and p-coumaryl alcohol lignin monomer interact with residues Ser163. **(E)** Molecular docking between ZjPOD51 and coniferyl alcohol lignin monomer interact with residues Gln137. **(F)** Molecular docking between ZjPOD51 and sinapyl alcohol lignin monomer interact with residues Arg32, His36 and Ser163. The dashed line represents hydrogen bonds. CW, cell wall; PM, plasma membrane.

To better understand the function of ZjPOD51, the characteristics of the ZjPOD51 protein were analyzed. First, phylogenetic analysis revealed that ZjPOD51 is a Class III peroxidase (Fig. 3A). Multiple sequence alignment also showed that these proteins contained relatively conserved secretory peroxidase and ligninase domains (Fig. 3B), indicating that POD51 was conserved during plant evolution. The secondary structure of ZjPOD51 consisted of 75 alpha helices (22.94%), 66 extended strands (20.18%), and 186 random coils (56.88%) (Fig. S3A). In addition, it also had a hydrophobic transmembrane sequence (Fig. S3B), and its N-terminus contained a signal peptide of 27 residues that is required for correct trafficking to the apoplast (Fig. S3C).

Subcellular localization and plasmolysis experiments showed that the ZjPOD51-GFP fusion protein was located in the cell wall (Fig. 3C, Fig. S3D). Moreover, molecular docking indicated that ZjPOD51 could interact with three lignin monomers, p-coumaryl/coniferyl/sinapyl alcohol, via Ser163, Gln137, Arg32, and His36. Among them, sinapyl alcohol exhibited the strongest binding with ZjPOD51 (−6.9 kcal/mol), and there were three binding sites (Fig. 3D and F). Taken together, these results suggested that *ZjPOD51* is involved in the polymerization of lignin monomers in the cell wall.

### *ZjPOD51* upregulates lignin synthesis in jujube

**Figure 4:**
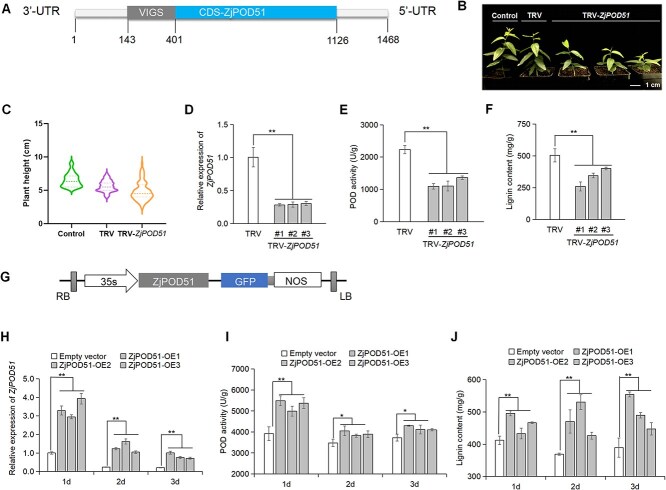
*ZjPOD51* upregulates lignin synthesis in jujube. (**A**) Gray box represents silent segment in ZjPOD51. (**B**) Phenotype of TRV-*ZjPOD51* lines (25-day-old seedlings), scale bar = 1 cm. (**C**) Comparison of plant heights between control, TRV, and TRV-Z*jPOD51* lines. (**D**–**F**) Expression levels, POD enzyme activity, and lignin contents of TRV and TRV-*ZjPOD51* lines. Three silenced lines were named #1, #2, and #3. (**G**) Schematic diagram of *ZjPOD51* constructs. LB, RB, 35S, ZjPOD51, GFP, and NOS indicate the T-DNA left border, T-DNA right border, cauliflower mosaic virus (CaMV) 35S promoter, *ZjPOD51* ORF, GFP tag, and the CaMV 35S terminator, respectively. (**H**–**J**) Expression levels, POD enzyme activity, and lignin contents in jujube fruits with transient overexpression of *ZjPOD51* at 1, 2, and 3 days after treatment. Bars represent the mean values ± SEs (*n* = 3). ** represents *P* < 0.01, * represents *P* < 0.05.

To verify the function of *ZjPOD51*, it was silenced in jujube seedlings by a TRV-based virus-induced silencing (VIGS) assay (Fig. 4A). As shown in Fig. 4B and C, compared with the control plants, the *ZjPOD51*-silenced seedlings exhibited significant dwarfing symptoms. *ZjPOD51* expression in the TRV-*ZjPOD51* lines was significantly reduced (Fig. 4D). Accordingly, the activities of POD and the lignin content in the silenced lines were significantly lower than those in the controls (Fig. 4E and F). Moreover, the transient overexpression of *ZjPOD51* in jujube fruits was performed (Fig. 4G and H). The POD activity and lignin content in the *ZjPOD51*-OE lines were markedly greater than those in the controls (Fig. 4I and J). Overall, the above results indicated that *ZjPOD51* positively regulates lignin synthesis in jujube.

### *ZjPOD51* positively regulates jujube defense response against phytoplasma

**Figure 5:**
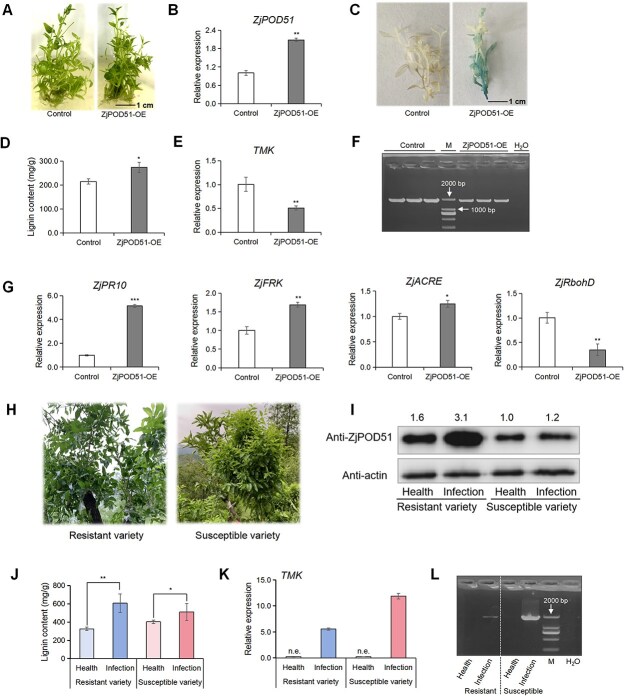
*ZjPOD51* upregulates lignin synthesis and triggers immune response in jujube after phytoplasma infection. (**A**) Phenotype of diseased jujube seedlings after transient overexpression of *ZjPOD51*. (**B**) Expression of *ZjPOD51* in transient overexpression lines. (**C**) Identification of positive transgenic seedlings by GUS staining. (**D**) Lignin content in *ZjPOD51*-OE lines. (**E**) The *TMK* expressions in control and *ZjPOD51*-OE lines. (**F**) The phytoplasma identification in control and *ZjPOD51*-OE lines by P1/P7 primer pair. Note: M indicates DL 2000 marker, the 1.8-kb band was a target band, H_2_O was used as a negative control. (**G**) Expression of defense-related genes in *ZjPOD51*-OE lines. (**H**) Symptoms in JWB-resistant and susceptible varieties after phytoplasma infection (in August). (**I**) Western blotting (WB) results of ZjPOD51 (35 kDa) in JWB-resistant and susceptible varieties. The quantitative analysis of WB bands was performed by Image J software, and the data were homogenized. (**J**) Lignin content in JWB-resistant and susceptible varieties. (**K**) The *TMK* expression in JWB-resistant and susceptible varieties, n.e. represents no expression. (**L**) The phytoplasma identification in JWB-resistant and susceptible varieties by P1/P7 primer pair, the 1.8-kb band was a target band.

To investigate whether *ZjPOD51* contributes to the jujube defense response against phytoplasma, *ZjPOD51* was transiently overexpressed in JWB-infected seedlings (Fig. 5A). Compared to that in the control, *ZjPOD51* expression increased significantly in the OE lines (Fig. 5B). The GUS (β-glucuronidase) staining assay also showed that *ZjPOD51* was overexpressed in diseased seedlings (Fig. 5C). The lignin content in the *ZjPOD51-OE* lines increased, and the expression of the phytoplasma marker gene *TMK* significantly decreased (Fig. 5D and E), indicating that the phytoplasma concentration had decreased. The universal phytoplasma-specific primer set P1/P7 for the 16S rDNA sequence was also used for phytoplasma identification (Fig. 5F). Further analysis revealed that the expression of defense-related genes was also triggered in the *ZjPOD51-OE* lines (Fig. 5G). Taken together, these results suggested that the overexpression of *ZjPOD51* improved the jujube defense response against phytoplasma.

Next, the JWB-resistant and susceptible varieties were applied and infected by grafting on JWB-infected jujube trees (Fig. 5H). After phytoplasma infection, the phloem proportion, ZjPOD51 expression, and lignin content in the resistant variety significantly increased compared to those in the susceptible one (Fig. S4C and D, Fig. 5I and J). *TMK* expression and P1/P7 identification revealed that the phytoplasma concentration in the resistant variety was low (Fig. 5K and L), suggesting that its defense response against phytoplasma was greater than that of the susceptible variety. This result was consistent with the overexpression of *ZjPOD51* in JWB-infected seedlings, i.e. the high expression of *ZjPOD51* could contribute to jujube defense response against phytoplasma.

### *ZjPOD51* enhances lignin synthesis and improves disease defense in *Arabidopsis*

**Figure 6:**
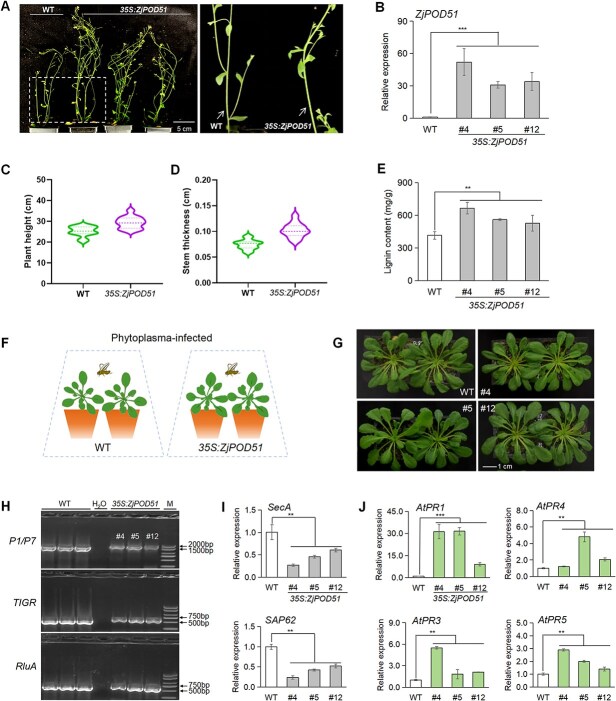
Overexpression of *ZjPOD51* in *Arabidopsis* improved the lignin synthesis and defense response against phytoplasma. (**A**) Phenotype of 35S:*ZjPOD51* transgenic plants. (**B**) Expression of *ZjPOD51* in three transgenic lines (#4, #5, and #12). (**C**) Comparison of plant heights between WT and transgenic lines. (**D**) Comparison of stem thickness between WT and transgenic lines. (**E**) Lignin contents of WT and transgenic lines. (**F**) *Arabidopsis* infected with LCLRD-phytoplasma by leafhoppers. (**G**) Phenotypic observation of WT and transgenic lines inoculated with phytoplasma. (**H**) The phytoplasma identification in WT and *ZjPOD51*-OE lines by three primer pairs. The primers of P1/P7, *TIGR*, and *RluA* for the 16S rDNA sequence were employed for phytoplasma identification using PCR. M indicates DL 2000 marker, the bands of 1.8 Kb, 706, and 778 bp were target bands, respectively. H_2_O was used as a negative control. (**I**) The *SecA* and *SAP62* expressions in WT and *ZjPOD51*-OE lines using qRT-PCR. (**J**) Expression of *AtPRs* in WT and transgenic lines after phytoplasma inoculation.

To further elucidate the function of *ZjPOD51*, it was ectopically overexpressed in *Arabidopsis*. Three transgenic lines were selected, and their stems were thicker than those of the wild-type (WT) plants (Fig. 6A–D). Compared with the controls, the lignin content of the transgenic lines significantly increased (Fig. 6E), indicating that *ZjPOD51* upregulates lignin synthesis.

To investigate the phytoplasma defense response of *ZjPOD51*, WT and *ZjPOD51-OE* transgenic *Arabidopsis* plants were inoculated with lettuce chlorotic leaf rot disease (LCLRD) phytoplasma by leafhoppers (Fig. 6F). After 18 days of cultivation, the growth status of *Arabidopsis* infected with phytoplasma is shown in Fig. 6G, and the phytoplasma was detected in both WT and *ZjPOD51-OE* lines (Fig. 6H). Subsequently, the expression of phytoplasma *SecA* and *SAP62* revealed that the phytoplasma concentration in the *ZjPOD51- OE* lines was low (Fig. 6I), suggesting that their defense response against phytoplasma was greater than that of the WT ones. And the expression of pathogenesis-related genes (*PRs*) increased significantly in the *ZjPOD51-OE* lines (Fig. 6J). Thus above results indicated that the high expression of *ZjPOD51* could contribute to *Arabidopsis* defense response against phytoplasma.

Besides, WT and *ZjPOD51-OE* transgenic *Arabidopsis* plants were inoculated with *P. syringae pv. tomato* DC3000 (*Pst*. DC3000). At 48 h postinoculation, the symptoms in the WT plants were more severe than those in the transgenic plants (Fig. S5A). Bacterial growth decreased in the transgenic lines compared to that in the WT ones (Fig. S5B). The expression of *PRs* also increased significantly in the *ZjPOD51-OE* lines (Fig. S5C). Therefore, a stronger defense response was triggered in *ZjPOD51-OE* lines after inoculation with *Pst.* DC3000, suggesting that *ZjPOD51* also could enhance the basal immunity of *Arabidopsis*.

### ZjMYB44 upregulates *ZjPOD51* expression by directly binding to its promoter

**Figure 7:**
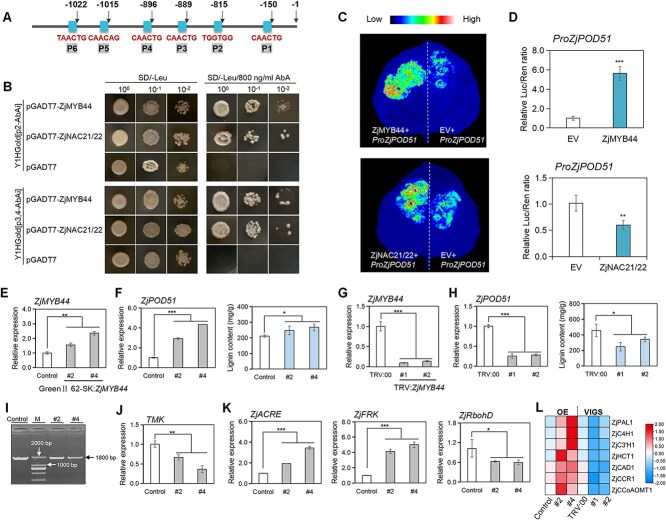
*ZjMYB44* improves defense response to phytoplasma by activating *ZjPOD51* and related genes in lignin synthesis. (**A**) Six MYB-binding sites of the *ZjPOD51* promoter. (**B**) Y1H assay validation of pGADT7-ZjMYB44, pGADT7-ZjNAC21/22 binding to *ZjPOD51* promoter. Screening results of AbA inhibitory concentration are shown in Fig. S7. (**C**) Dual luciferase reporter (DLR) assays to identify the activation of *ZjMYB44* and *ZjNAC21/22* on the promoters and repressor of *ZjPOD51*. EV represents the empty vector and was used as the negative control. (**D**) Luciferase activities in tobacco leaf cotransformed with the constructs were presented as the ratio of *LUC* activity to *REN* activity. The average value of fluorescence in EV was set as 1. Bars represent the mean values ± SEs (*n* = 6). (**E**) Expression of *ZjMYB44* in *ZjMYB44-OE* lines. Two OE plants were named #2 and #4. (**F**) Expression of *ZjPOD51* and lignin content in *ZjMYB44-OE* lines. (**G**) Expression of *ZjMYB44* in *ZjMYB44-*silenced lines. Two silenced plants were named #1 and #2. (**H**) Expression of *ZjPOD51* and lignin content in *ZjMYB44-*silenced lines. (**I**) The phytoplasma identification in *ZjMYB44-OE* lines by P1/P7 primer pair. (**J**) *TMK* expression in *ZjMYB44-OE* lines by qRT-PCR. (**K**) Expression of defense-related genes in *ZjMYB44-OE* seedlings. (**L**) Expression levels of related genes in lignin synthesis in *ZjMYB44-OE* and *ZjMYB44-*silenced lines. The red square represents high abundance expression, and the blue represents low abundance expression. The raw data in Fig. 7L are shown in Table S3.

To explore the regulatory factors of *ZjPOD51*, its promoter sequence was cloned and analyzed, and six MYB TF binding elements were found (Fig. 7A, Table S2). Moreover, cDNA library screening was carried out, and several MYB and NAC TFs were identified. Yeast one-hybrid (Y1H) and dual-luciferase (LUC) assays revealed that *ZjMYB44* directly activates *ZjPOD51* transcription (Fig. 7B–D). The expression of *ZjMYB44* in jujube significantly increased after phytoplasma infection, and it was also positively correlated with *ZjPOD51* expression (Fig. S6B and C). Phylogenetic analysis indicated that ZjMYB44 was closely related to AtMYB44 (Fig. S6D), which is an integral component of the PTI pathway and confers disease resistance [30].

Next, the disease resistance of *ZjMYB44* was further verified. Overexpression of *ZjMYB44* in JWB-infected seedlings upregulated *ZjPOD51* expression and promoted lignin content (Fig. 7E and F). On the contrary, *ZjPOD51* expression and the lignin content in the *ZjMYB44*-silenced lines were significantly reduced (Fig. 7G and H). Further analysis shown that overexpression of *ZjMYB44* in JWB-infected seedlings reduced the phytoplasma content (Fig. 7I and J). Moreover, the defense-related genes, *ZjACRE* and *ZjFRK*, were upregulated, but the expression of *ZjRbohD*, which is related to ROS production, decreased (Fig. 7K). In addition, several genes involved in lignin biosynthesis were upregulated in the *ZjMYB44-OE* lines but downregulated in the silenced lines (Fig. 7L, Fig. S6A, Table S3), indicating that ZjMYB44 might target multiple genes involved in lignin synthesis.

### The ZjMYB44-ZjPOD51 module was responsive to H_2_O_2_

**Figure 8:**
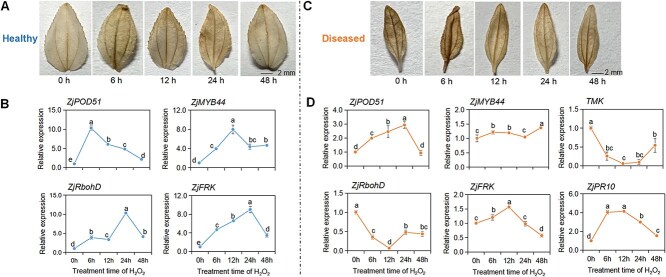
ZjMYB44-ZjPOD51 module was responsive to H_2_O_2_. (**A**, **C**) H_2_O_2_ detection in jujube seedlings by 3′3′-diaminobenzidine (DAB) staining after H_2_O_2_ treatment. (**B**) Expression level of *ZjPOD51* and related genes in healthy seedlings after H_2_O_2_ treatment. (**D**) Expression level of *ZjPOD51* and related genes in JWB-diseased seedlings after H_2_O_2_ treatment. Duncan’s multiple range test was used for statistical analysis, different letters indicate significant differences, while the same letters indicate no significant differences.

To determine whether H_2_O_2_ can induce the expression of *ZjPOD51* and *ZjMYB44*, healthy jujube seedlings were sprayed with H_2_O_2_ (Fig. 8A). The expression of *ZjPOD51*, *ZjMYB44*, and defense-related genes were induced by H_2_O_2_ (Fig. 8B), which may be related to the antioxidant and detoxification effects of these genes. In a previous study, the H_2_O_2_ content increased, and H_2_O_2_ accumulated in jujube after phytoplasma infection [27]. Meantime, JWB-infected seedlings were also treated with H_2_O_2_ (Fig. 8C). H_2_O_2_ treatment promoted the expression of *ZjPOD51* and *ZjMYB44* and significantly decreased the phytoplasma content (Fig. 8D). The expression of defense-related genes, *ZjFRK* and *ZjPR10*, also increased after treatment. *ZjRbohD* showed the opposite trend compared to that of *ZjPOD51* (Fig. 8D). These results indicated that the expression of the ZjMYB44-ZjPOD51 module was induced by H_2_O_2_ accumulation caused by phytoplasma infection.

## Discussion

### *ZjPOD51* enhances jujube defense response against phytoplasma by upregulating lignin biosynthesis

Pathogen infection typically stimulates phenylpropanoid metabolism in plants to improve host defense response [31], and major antimicrobial substances such as phenolic compounds, lignin, and flavonoids are synthesized by this pathway [32, 33]. Phytoplasmas only parasitize plant phloem tissue, thus the phloem-enriched bark of jujube plants was used as the test material in this study. The flavonoid content increased after jujube was infected by the phytoplasma, but the increase in lignin content was more significant (Fig. 1B, Fig. S1E–G), especially for G-type and S-type lignin (Fig. 1C). In flax, after inoculation with *Fusarium oxysporum*, the S-type lignin content in the cell wall increased 36-fold, and the S/G lignin ratio increased 2-fold [34]. In sea-island cotton, after the occurrence of *Verticillium* wilt, the G lignin monomer content significantly increased [35]. In *Arabidopsis*, the defense-induced lignin is composed mainly of G-type lignin [36]. In summary, for most plant species, G-type and S-type lignin may play the main role in defense response. In this study, the contents of G-type and S-type lignin also increased in the jujube phloem infected by phytoplasmas.

The genes involved in lignin synthesis pathways, such as those encoding PODs, usually change in plants under pathogen infection. *HvPOD40* and *TaPOD10* enhance wheat defense response against *Blumeria graminis* [16, 37, 38]. *CsPOD25* provides resistance to citrus bacterial canker by maintaining ROS homeostasis and cell wall lignification [17]. Here, *ZjPOD51* was also induced in jujube phloem by phytoplasma stress (Fig. 2C), and its function in lignin synthesis was verified. Moreover, the overexpression of *ZjPOD51* in diseased jujube seedlings strongly reduced the phytoplasma content, indicating that it could confer defense response against phytoplasma (Fig. 5). This result was further confirmed by the increased levels of the ZjPOD51 protein in the resistant variety after phytoplasma infection (Fig. 5).

### *ZjMYB44* might be a positive regulator of the entire lignin biosynthesis pathway

*MYB44* is a multifunctional transcription activator involved in phytohormone signaling pathways, such as those involving JA, SA, abscisic acid (ABA), and ethylene, and responds to biotic and abiotic stresses, such as fungal, microbial, drought, and salt stresses [21, 39, 40]. *AtMYB44* regulates SA- and JA-mediated defense responses by directly regulating the expression of *WRKY70* [21]. Recent studies have shown that *MYB44* is an essential component of the PTI pathway, conferring disease resistance by increasing the expression of *EIN2*, *MPK3*, and *MPK6* [30]. The synthesis of lignin-mediated MAPK pathway may be closely associated with the disease resistance of bitter gourd [41]. In this study, several genes involved in lignin biosynthesis were upregulated in the *ZjMYB44-OE* lines but downregulated in the silenced lines (Fig. 7L), Therefore, *ZjMYB44*, as a novel positive regulator, not only regulates the expression of *ZjPOD51* but may also affect multiple genes involved in lignin synthesis, and enhancing jujube defense response against phytoplasma.

### ZjMYB44-ZjPOD51 enhances H_2_O_2_-mediated defense to the phytoplasma in jujube

As an important class of signaling molecules, ROS not only can kill pathogens, but also participate in cell wall remodeling, hypersensitivity, and the occurrence of systemic acquired resistance to resist the invasion of pathogens [42–45]. Among ROS, H_2_O_2_ can enhance plant cell wall by influencing lignin synthesis through regulation of POD [46], thereby limiting pathogenic bacteria to the site of infection [47]. Our previous research showed that H_2_O_2_ should be an important signaling molecule that contributes to the jujube defense to phytoplasma [48]. In this study, H_2_O_2_ treatment in diseased jujube seedlings could increase the expression of *ZjMYB44* and *ZjPOD51* (Fig. 8). And, the increasing expression of *ZjPOD51* could reduce the expression level of *ZjRbohD* (Figs 7K and 8D), which is involved in ROS production. It is reported that POD protein functions as lignin biosynthesis factor and ROS clearer [17, 49]. But whether *ZjPOD51* could feedback regulate ROS production still needs further study.

**Figure 9:**
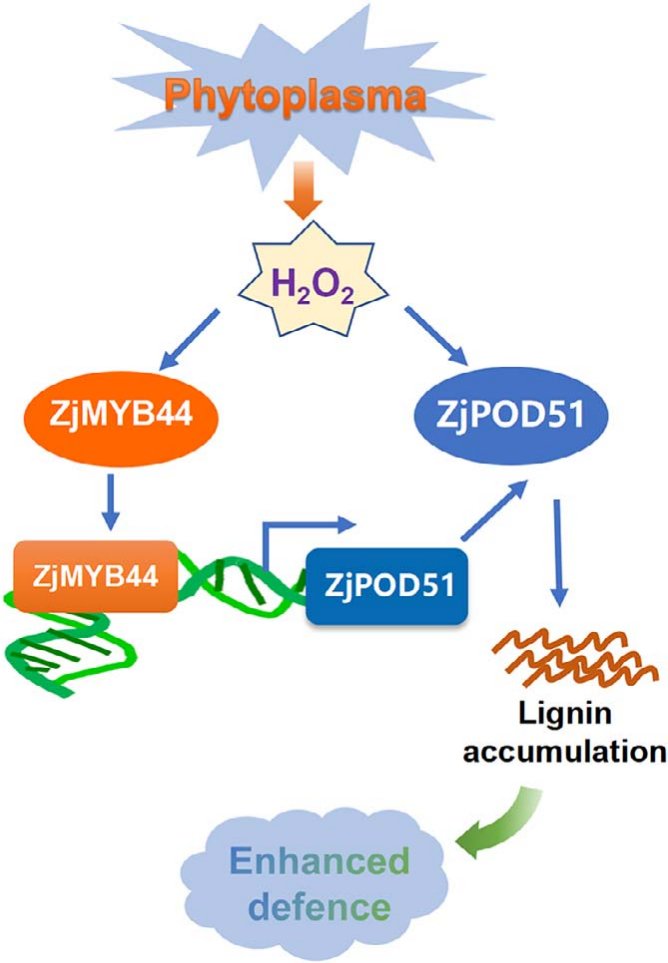
The ZjMYB44-ZjPOD51 module enhances H_2_O_2_-mediated defense response against phytoplasma by activating lignin synthesis.

As vital enzymes for ROS homeostasis, Class III PODs are postulated to be major regulators of extracellular H_2_O_2_ and O_2_^.-^ levels depending on the cycle involved, i.e. the peroxidative cycle (ROS scavenging) or hydroxylic cycle (ROS production) [50]. A recent study revealed that citrus huanglongbing (HLB) is an immune-mediated disease that can be mitigated with antioxidants [51]. In this study, the expression of *ZjPOD51* was strongly induced by H_2_O_2_, stimulating POD activity to promote ROS clearance. After phytoplasma infection, ROS are produced, and H_2_O_2_ can act as a signaling molecule to activate *ZjMYB44* and *ZjPOD51* and then promote lignin accumulation, leading to enhanced jujube defense response against phytoplasma (Fig. 9). At the same time, many plant hormones such as JA and SA are also involved in the defense process. In a previous study, phytoplasma infection could amplify the accumulation of JA and antagonize the SA accumulation in jujube [48]. Exogenous SA and JA application improved tomato resistance to TYLCV (Tomato yellow leaf curl virus) infection while increasing H_2_O_2_ accumulation. [52]. Thus the crosstalk regulation among ROS, JA/SA, and lignin in jujube trees after phytoplasma infection needs in-depth study. In short, this study provides new insights into the molecular mechanisms by which ROS, lignin, and ZjMYB44-ZjPOD51 regulate jujube–phytoplasma interactions and provides potential clues for breeding resistant jujube plants.

## Materials and methods

### Plant materials

‘Pozao’ (*Z. jujuba* Mill.) is a typical susceptible cultivar to JWB phytoplasma. After infection, the trees develop serious symptoms, including phyllody and witches’ broom. Healthy and JWB-infected ‘Pozao’ trees were used in the test. Three plants in similar growth states served as biological duplicates. These trees were cultured at the Experimental Station in Fuping County, Hebei Province, and cultivated under natural environmental conditions. The annual secondary branches of the jujube trees were chosen, the outermost layer of the periderm tissue was removed, and the phloem was maintained and collected from June to September (Fig. S1A).

Leaf types reflecting different degrees of JWB infection, namely, apparently normal leaves (ANL), witches’ broom leaves (WBL), and phyllody leaves (PL), were collected from infected trees, and healthy leaves (HL) were collected from healthy trees.

Scions of the JWB-resistant variety ‘T13’ and susceptible variety ‘Dongzao’ were grafted onto JWB-infected and healthy trees [27, 53]. Grafting was performed in May, and 5 g of newly grown phloem from the scion was taken for testing in July. Each treatment has three biological replicates. After collection, all samples were quickly frozen in liquid nitrogen and stored at −80°C for RNA extraction and further testing.

### Detection of the JWB phytoplasma

The 16S rDNA sequence is the most widely utilized region for identifying phytoplasma. The universal phytoplasma-specific primer set P1/P7 for the 16S rDNA sequence was employed for phytoplasma identification using polymerase chain reaction (PCR) [54, 55]. The *thymidylate kinase* gene (*TMK*, KC493615.1) is a marker gene used to identify JWB phytoplasma [56]. The expression of the *TMK* gene in jujube samples displaying disease symptoms was examined by quantitative real-time PCR (qRT-PCR), with *ZjACT* as the internal control [57]. All treatments included three biological replicates.

### Total DNA, RNA extraction, and qRT-PCR analysis

To detect phytoplasma, total genomic DNA was extracted from samples via the cetyltrimethylammonium bromide (CTAB) technique [58], and the PCR mixture was processed via the method described [55]. The RNAprep Pure Plant plus Kit (Tiangen, China) was used for RNA isolation, then cDNA was synthesized using FastQuant RT Super Mix Kit (Tiangen, China). The qRT-PCR analysis was performed using the StepTwoPlus Real-Time PCR System according to the manufacturer’s instructions (Sanshi, China). *ZjACT* was used as internal control [57]. Relative expression levels were calculated by the 2^-△△CT^ method [59]. Gene-specific primers are shown in Table S4.

### Metabolite extraction and HPLC-MS/MS analysis

The freeze-dried healthy and diseased phloems were crushed using a mixer mill (MM 400, Retsch) in zirconia beads at 30 Hz for 1.5 min. The 0.1 g powder was weighed and extracted overnight at 4°C in 0.6 ml of 70% aqueous methanol. After centrifugation at 10 000 g for 10 min, the extract was filtered. The liquid chromatography electrospray ionization tandem mass spectrometry (LC-ESI-MS/MS) system was used to perform relative quantification of small-molecule metabolites in lignin biosynthesis by Metware Biolab (Wuhan) Co. Ltd. The specific parameters refer to the relevant references [60].

### Determination of POD activity and lignin, flavonoid, and cellulose contents

According to the instructions [61], the ELISA Kits of plant peroxidase (PAL, 4CL, CAD, CCR, etc.) and lignin (flavonoid, cellulose, hemicellulose) (Meimian, China) were used to determine the indicators. Three biological replicates were performed for each treatment.

### Subcellular localization

The fusion vector CaMV35S-ZjPOD51-GFP was introduced into *Agrobacterium tumefaciens* GV3101, infiltrated into the leaves of *Nicotiana benthamiana* and onion epidermal cells. The method for infecting onions was performed as previously described [62]. The tobacco plants were cultured in the dark for 24 h and then transferred to a chamber (16 h/8 h, 25°C/18°C, light/dark). The onions were cultured in the dark for 48 h at 28°C. At 48 h after transient transformation, the infected tobacco leaves and onion epidermis were subjected to confocal laser microscopy (LSM800; Zeiss) to monitor green fluorescent protein (GFP) fluorescence.

### Transient overexpression of *ZjPOD51*

The coding sequence (CDS) of *ZjPOD51* was cloned and inserted into the pCambia1300 vector and then transferred into the GV3101 strain. Transient overexpression in jujube fruits was performed as previously described [61]. The injected jujube fruits were sampled on the first, second, and third days to analyze gene expression, POD activity, and lignin content.

In addition, the full-length CDS of *ZjPOD51* was cloned into the pGreen 62-SK overexpression vector and then transferred into the GV3101 strain. After being subcultured for 15 days, diseased seedlings with heights ranging from 4.0 to 5.0 cm were transformed. The infection process was as follows: the jujube seedlings were placed into a 50 ml sterile syringe and completely immersed in 15–20 ml of infiltration buffer [the suspension was prepared in Murashige and Skoog (MS) basic liquid culture medium with acetosyringone (200 mM), pH [5.6–5.8] to an OD_600_ of 0.6–0.8. By pulling the piston, the inside of the syringe was kept in a negative pressure vacuum state for 10 min, the piston was released, and the infection solution entered the jujube tissue under pressure. Finally, the infected tissue was placed on filter paper to dry and subcultured on MS solid culture medium for further cultivation. Jujube plants infected by *A. tumefaciens* were placed in the dark for 24 h and then cultivated under light for 14 days at 25°C. After confirming the positive plants via qRT-PCR, subsequent experiments were conducted. The transient overexpression of *ZjMYB44* follows the above method. The primers used are shown in Table S4.

### TRV-based virus-induced silencing of *ZjPOD51 / ZjMYB44*

Approximately 250 bp of *ZjPOD51* was cloned into the pTRV2 vector, and the pTRV2 and pTRV2-*ZjPOD51* plasmids were subsequently transformed into the GV3101 strain. The infection steps were performed according to the method reported [61, 63]. After 25 days of culture, the height, POD activity, and lignin content of the transformed plants were tested. Each treatment was evaluated with 24 jujube seedlings. The pTRV1:pTRV2 mixed vector served as the control. The primers used are listed in Table S4. The silencing of *ZjMYB44* follows the above method.

### Bioinformatics analysis

The secondary structure of ZjPOD51 was predicted according to the ExPASy Proteomics Server. The protein transmembrane structure was analyzed by TMHMM software. The signal peptide was predicted via SignalP 6.0. The phylogenetic tree was constructed using MEGA 7.0. To assess the interactions of the three lignin monomers with ZjPOD51, molecular docking was performed. For molecular docking, proteins were prepared using Alphafold (https://alphafold.ebi.ac.uk/). The ligand was prepared using PubChem (https://pubchem.ncbi. nlm.nih.gov/). Analysis of docking conformations was performed with Pdbqt files and visualized in PyMol [64, 65].

### Organizational observation

A paraffin section assay was used to observe the growth of phloem. For the preparation and observation of paraffin sections, bearing branches <0.5 cm in length were sliced from healthy and diseased jujube trees of ‘Pozao’ in July, fixed with AAF fixative (Solarbio, Beijing, China), dehydrated, paraffin-embedded, sectioned, stained, sealed, and air-dried for observation. Tissue cell images were analyzed utilizing imaging software (ZP-1000 microscope, PuZhe, Shanghai, China).

Lignin deposition was visualized by Wiesner reagent using histochemical staining of hand-cut cross-sections from jujube stems [66]. The test sample was treated with 2% phloroglucinol in 95% ethanol solution for 5 min and mounted in 6 M HCl to determine the presence of lignin [67].

### GUS staining assay

For GUS staining, 14-day-old diseased seedlings with gene overexpression were stained with 5-bromo-4-chloro-3-indolyl glucuronide at 37°C for 12 h as described [68].

### Protein extraction, western blotting, and antibodies

A total of 0.5 g of healthy and phytoplasma-stressed phloem tissue from the resistant and susceptible varieties was quickly frozen and ground to powder in liquid nitrogen. Protein extracts were treated according to the method as reported [69]. The protein expression and purification of ZjPOD51 were performed as described previously [70]. The anti-ZjPOD51 antibody (Catalog no. CM0309) was customized by PTM BioLabs (Hangzhou, China). Antiactin (plant) Mouse mAb (Catalog no. PTM6702, PTM BioLabs) was used as plant internal reference in this study.

### Genetic transformation and *Pst.* DC3000 treatment

All *Arabidopsis thaliana* plants used in the study were of Columbia ecotype background. The fusion vector CaMV35S-ZjPOD51-GFP was introduced into *Agrobacterium* strain GV3101 and infiltrated into *Arabidopsis* plants via the floral dip method. First-generation seeds of the *ZjPOD51* transgenic plants were chosen by MS medium containing hygromycin B (50 mg/l). Using qRT-PCR, the transgenic plants were verified, and three generations of homozygous lines were selected.

The pathogen *Pst*. DC3000 was cultured in KB medium with rifampicin (50 mg/l) until the OD_600_ of 1.0. Then bacterial fluids were collected, centrifuged, and resuspended in sterile 10 mM MgCl_2_ buffer to the OD_600_ of 0.001. Next, the bacterial cells were inoculated into the 4-week-old WT and *ZjPOD51-OE Arabidopsis* rosette leaves via a needleless 1 ml syringe. After 48 h, leaves from inoculated plants were collected via a punch (6 mm) and homogenized via MgCl_2_ solution. Subsequently, the bacterial diluents at different concentrations were spread on KB medium with rifampicin (50 mg/l), the plates were incubated at 28°C for 48 h, and bacterial colonies were counted for the appropriate concentration [71, 72]. Finally, the remaining leaves were taken for RNA extraction to detect the expression of *AtPRs*.

### Phytoplasma infection assays

For phytoplasma inoculation, the method refers to previous study [73]. In brief, 4-week-old WT and *ZjPOD51* transgenic *Arabidopsis* plants were grown under short-day conditions (10 h light/14 h dark, 22°C). Two plants as one group were exposed to six male leafhoppers carrying LCLRD-phytoplasma in a net bag for 4 days, then the leafhoppers were removed. A total of 24 WT and 24 transgenic plants were used in phytoplasma inoculation. After 18 days of growth, DNA and RNA were collected from the newly grown leaves of infected *Arabidopsis* to detect phytoplasma and the expression of *AtPRs*.

The universal phytoplasma-specific primers of *TIGR* and *RluA* for the 16SrDNA sequence were employed for LCLRD-phytoplasma identification using PCR [74]. The S*ecA* and *SAP62* were marker genes used to detect phytoplasma concentration by qRT-PCR [75].

### Yeast one-hybrid assay

A Y1H assay was used to investigate the interaction of *ZjPOD51* promoter with its supposed target gene. The *ZjPOD51* promoter fragments were divided into two sections: P2 and P3–4. The sequences of P2 and P3–4 are shown in Table S4. The two DNA fragments were inserted into the pAbAi vector. The full-length CDSs of *ZjMYB44* or *ZjNAC21/22* were cloned into the pGADT7 vector. Both fusion constructs were cotransformed into to the Y1H Gold strain and cultured on SD/−Leu/−Ura screening medium. Aureobasidin A (AbA) was used to evaluate the interactions between the *ZjPOD51* promoter and *ZjMYB44* or *ZjNAC21/22*.

### Dual-luciferase assay

The *ZjPOD51* (1000 bp upstream of ATG) promoter fragment was cloned and inserted into the pGreenII-LUC vector. The CDSs of *ZjMYB44* or *ZjNAC21/22* were cloned and inserted into pGreenII 62-SK vector. The primers used to construct the vectors are listed in Table S4. Recombinant plasmids were transformed into *Agrobacterium* strain GV3101. *Agrobacterium* harboring recombinant plasmids were infiltrated into 4-week-old tobacco leaves. Tobacco leaves were transiently expressed as described [76].

LUC/REN (Renilla) activity was analyzed using the Dual-Luciferase Reporter Assay System (11402ES60, Yeasen Biotechnology, Shanghai) according to the instructions. Six independent biological replicates were analyzed.

### H_2_O_2_ treatment

Seedlings of the jujube variety ‘Dongzao’ were cultured at the Research Center of Chinese Jujube, Hebei Agricultural University. After being subcultured for 15 days, the diseased seedlings with heights ranging from 4.0 to 5.0 cm were subjected to foliar spraying of 1 mmol l^−1^ H_2_O_2_. The treatment concentration was based on the result of pre-experiment and slightly adjusted according to the described method [77, 78]. After 6, 12, 24, and 48 h, the seedlings were removed for RNA extraction and staining observation. Three biological replicates were collected for each treatment.

The seedlings were stained with 3′-3′-diaminobenzidine (DAB) to detect H_2_O_2_. The seedlings were vacuum infiltrated with DAB (1 mg ml^−1^, pH 3.8) solution at 25°C in darkness for 6 h. The dyed seedlings were boiled in 95% ethanol until chlorophyll was removed for imaging.

### Statistical analysis

Differences between two groups were detected using the *t*-test (*P < 0.05; **P < 0.01; ***P < 0.001). Different lowercase letters denote significant differences between more than two groups of data at *P* < 0.05 based on one-way analysis of variance. Correlations were calculated using IBM SPSS Statistics, and visualized on heatmaps using TBtools.

## Supplementary Material

Web_Material_uhaf083

## Data Availability

The data that support the findings of this study are available in the supplementary material of the article.
